# Enhanced Visible-Light Photocatalytic Activity of Ag QDs Anchored on CeO_2_ Nanosheets with a Carbon Coating

**DOI:** 10.3390/nano9111643

**Published:** 2019-11-19

**Authors:** Xiaogang Zheng, Qian Chen, Sihao Lv, Xiaojin Fu, Jing Wen, Xinhui Liu

**Affiliations:** 1 Research Center for Eco-Environmental Engineering, Dongguan University of Technology, Dongguan 523808, China; zhengxg123456@163.com; 2 College of Chemistry and Chemical Engineering, Neijiang Normal University, Neijiang 641100, China; cq18140267436@163.com (Q.C.); fu-xj2007@163.com (X.F.); 3 Key Laboratory of Comprehensive and Highly Efficient Utilization of Salt Lake Resources, Key Laboratory of Salt Lake Resources Chemistry of Qinghai Province, Qinghai Institute of Salt Lakes, Chinese Academy of Sciences, Xining 810008, China; 4 State Key Laboratory of Water Environment Simulation, School of Environment, Beijing Normal University, Beijing 100875, China; xhliu@bnu.edu.cn

**Keywords:** Ag QDs, CeO_2_ nanosheets, carbon coating, photocatalytic activity, visible light

## Abstract

Ag quantum dots (QDs) anchored on CeO_2_ nanosheets with a carbon coating (Ag/CeO_2_@C) (composites) were prepared via an in situ reduction approach for the photocatalytic degradation of Cr(VI) and tetracycline hydrochloride (TCH) in the visible-light region. The photocatalytic activity of Ag/CeO_2_@C was greatly affected by carbon content, Ag-doping content, Cr(VI) concentration, pH value, and inorganic ions. Enhanced photocatalytic activity was obtained by Ag/CeO_2_@C (compared to CeO_2_ and CeO_2_@C), of which 3-Ag/CeO_2_@C-2 with an Ag-doping content of 5.41% presented the best removal efficiency and the most superior stability after five cycles. **·**O_2_^−^ and **·**OH radicals were crucial for the photocatalytic capacity of 3-Ag/CeO_2_@C-2. The combined effect of the surface plasma resonance (SPR) of Ag QDs, an electron trapper of carbon shells, and the redox activity of the Ce(III)/Ce(IV) coupling induced efficient charge transfer and separation, suppressing the recombination of electron–hole pairs.

## 1. Introduction

The wide-ranging use of antibiotics, dyes, and heavy metals and their reckless release in water has drawn intensive attention due to their toxicity and non-biodegradability [1,2,3]. In recent years, many strategies, such as electrochemical oxidation [4], coagulation and flocculation [5], adsorption [6], membrane filtration [7], and advanced oxidation [8], have been applied for water purification. Photocatalysis, a typical advanced oxidation technique, has become the route with the most potential to conquer these intensifying environmental problems via the utilization of solar light [9,10,11]. Hence, various semiconductors, such as g-C_3_N_4_, TiO_2_, ZnS, ZnO, CuS, and MoS_2_, have been confirmed as cost-effective photocatalysts for the heterogeneous photocatalytic purification of polluted water [12,13,14,15,16,17]. Due to their wide band gap, nontoxicity, and high stability, cerium dioxide (CeO_2_) and TiO_2_ have been widely applied in photocatalytic reactions [18,19,20]. In addition, CeO_2_ exhibits strong UV-light sorption capacity and a high resistance to photocorrosion [21].

The adsorption and desorption capacities of oxygen ions are critical for the catalytic process of CeO_2_. The oxygen storage capacity of CeO_2_ is greatly affected by the redox activity of the Ce(III)/Ce(IV) coupling, further depending on the type and content of oxygen vacancies in the lattice structure [22]. The defect structure of Ce^3+^–O_v_–Ce^4+^ (O_v_- oxygen vacancy) and the formation of Ce^3+^ ions induce a red shift in the band gap of CeO_2_ [23]. In fact, the crystal defect of CeO_2_ is greatly related to its crystal structure and morphologies. Various microstructures of CeO_2_ have been fabricated for practical applications via different approaches in previous works, including bowknot-like crystallites, nanocubes, X-architecture, nanopolyhedra, square-like nanoparticles, nanosheets, nano-octahedrons, ribbon-like nanofibers, urchin-like hierarchical structures, flower-like microspheres, and well-aligned nanorod arrays [24,25,26,27,28,29,30,31,32,33]. Unfortunately, CeO_2_ with indirect band gap energy (3.2 eV) is strictly limited in the photocatalytic system of the UV-light region. Hence, nonmetal or metal ions such as S, N, P, Er, Fe, Sm, and Y have been doped in CeO_2_ to extend light harvesting to the visible-light region, leading to enhanced photocatalytic activity [34,35,36,37,38,39,40]. Apart from a doping strategy, noble metals such as Au and Ag loaded on CeO_2_ have been confirmed to present much better photocatalytic activity [41]. In addition, the fabrication of heterojunctions coupled with other semiconductors can simultaneously enhance the separation efficiency of charge carriers and restrain the recombination rate of photoexcited electron–hole pairs through the interface structure of different semiconductors, especially core–shell structures [42,43,44]. To further avoid photocorrosion in solar energy-driven reaction systems, carbon has been employed for the synthesis of heterojunction composites due to its unique physicochemical properties and low cost [45,46,47,48]. Carbon with a porosity structure and a high surface area not only exhibits excellent affinity for pollutant molecules, but also efficiently captures and transfers the photoexcited electron, leading to an enhancement in photocatalytic activity [49,50]. However, the combined effect of Ag doping and carbon coating on the enhanced photocatalytic capacity of CeO_2_ nanosheets has been scarcely reported in previous works.

This work focused on the effect of Ag quantum dots (QDs) on the photocatalytic activity of carbon-coated CeO_2_ (CeO_2_@C) nanosheets in the visible-light region. In this strategy, Ag QDs were anchored in situ on CeO_2_@C nanosheets to form Ag/CeO_2_@C. The photocatalytic capacity of Ag/CeO_2_@C was affected by the carbon dosage, the Ag-doping content, the Cr(VI) concentration, the pH value, and inorganic ions. The combined effects of the surface plasma resonance (SPR) of Ag QDs, an electron trapper of carbon shells, and the redox activity of the Ce(III)/Ce(IV) coupling were responsible for enhanced visible-light harvesting and efficient charge transfer and separation, leading to excellent photocatalytic activity in the CeO_2_ nanosheets [39,41,45,51,52]. The possible photocatalytic mechanism of Ag/CeO_2_@C is discussed in detail.

## 2. Materials and Methods

### 2.1. Preparation of Catalysts

CeO_2_ nanosheets were prepared via a hydrothermal route. Briefly, 1.0 mmol of cerium nitrate hexahydrate (Ce(NO_3_)_3_·6H_2_O) and 2 mmol of hexamethylenetetramine (C_6_H_12_N_4_) were dissolved in 70 mL of deionized water through vigorous stirring. Then, 3 mL of acetic acid (CH_3_COOH) was added to the above solution and stirred at room temperature for 2 h. This mixture was transferred to a 100 mL Teflon-lined autoclave and treated at 433 K for 9 h. After being cooled down to room temperature, the suspension was filtered, washed with ethanol and deionized water, dried at 333 K for 6 h, and calcined at 773 K for 3.0 h to obtain CeO_2_ nanosheets.

Carbon-coated CeO_2_ (CeO_2_@C) nanosheets were also synthesized through a hydrothermal route. Here, 0.1 g obtained CeO_2_ bulks, 0.2 g glucose (C_6_H_12_O_6_), and 1.0 g polyvinyl pyrrolidone (PVP, M = 58,000) were dispersed into 30 mL of deionized water through intensive stirring at room temperature for 2.0 h and then treated at 453 K for 15.0 h in a 50 mL Teflon-lined autoclave. After being cooled down to room temperature, the above suspension was centrifuged, washed, dried at 343 K for 5.0 h, and calcined at 773 K for 2.0 h in an N_2_ flow rate of 40 mL·min^−1^ to obtain CeO_2_@C nanosheets (called CeO_2_@C-1). With the above process, CeO_2_@C composites with varying carbon contents were obtained with different mass ratios of CeO_2_/glucose. CeO_2_@C-2 and CeO_2_@C-3 were obtained via the addition of glucose contents of 0.4 g and 0.6 g, respectively.

Ag QDs anchored in CeO_2_@C (Ag/CeO_2_@C) nanosheets were reduced in situ with the addition of sodium borohydride (NaBH_4_). In a typical process, 0.1 g CeO_2_@C bulks and 0.02 g AgNO_3_ were dispersed into 50 mL deionized water and then stirred at room temperature for 2.0 h. NaBH_4_ bulks of 0.05 g were added to the above solution through serious stirring at room temperature for 1.0 h. After the suspension was filtered, washed, and dried at 333 K for 6.0 h, Ag/CeO_2_@C nanosheets were obtained and called 1-Ag/CeO_2_@C. X-Ag/CeO_2_@C (X = 2, 3, and 4) nanosheets were respectively obtained via the addition of 0.03 g, 0.04 g, and 0.05 g AgNO_3_ in accordance with the above process.

### 2.2. Characterization of Catalysts

CeO_2_-based nanosheets were evaluated by X-ray diffraction (XRD, Bruker D8, Karlsruhe, Germany), inductively coupled plasma optical emission spectrometry (ICP-OES, Varian 710-ES, PaloAlto, CA, USA), X-ray photoelectron spectroscopy (XPS, Escalab 250, Waltham, MA, USA), physical adsorption (Quantochrome NOVA-2020, Boynton Beach, FL, USA), scanning electron microscopy (SEM, Hitachi S-3400, Tokyo, Japan), transmission electron microscopy (TEM, JEM-2010, Tokyo, Japan), high-resolution transmission electron microscopy (HRTEM, JEM-2100, Tokyo, Japan), Fourier-transform infrared spectra (FT-IR, Bruke Tensor 27, Karlsruhe, Germany), and UV–Vis diffuse reflectance spectra (UV-Vis DRS, Hitachi U-4100, Tokyo, Japan) (with BaSO_4_ as the reflectance standard and an integrated sphere attachment, Photoluminescence (PL, FLSP 920, Edinburgh, UK), with an excitation wavelength of 325 nm at room temperature and electron spin resonance (ESR, JES-FA200, Tokyo, Japan) with 5,5-Dimethyl-1-pyrroline N-oxide (DMPO) as spin trapping agent. In addition, photocurrents, Mott–Schottky curves, and the electrochemical impedance spectroscopy (EIS) of CeO_2_-based composites were evaluated in a three-electrode electrochemical workstation, which consisted of Pt film, KCl-saturated calomel, and FTO conductive glass (coated with 1 cm^2^ of CeO_2_-based composite films) serving as electrodes and 0.2 mol L^−1^ Na_2_SO_4_ serving as an electrolyte. The photoelectrochemical properties of CeO_2_-based composites were analyzed on a photoelectric instrument (CEL-PECX2000, Beijing CEL Tech. Co., Ltd., Beijing, China) equipped with a Vertex. C. EIS electrochemistry workstation (Ivium Technologies B.V., Eindhoven, Netherlands) and a visible-light source (an Xe lamp) at room temperature.

### 2.3. Photocatalytic Activity

CeO_2_-based composites were applied for the visible-light-driven photodegradation of Cr(VI) ions and tetracycline hydrochloride using an Xe lamp as a light source (300 W). In a typical process, 0.1 g CeO_2_-based bulks was dispersed into 100 mL of a potassium dichromate (K_2_CrO_4_) solution of 20 mg·L^−1^ (or a tetracycline hydrochloride solution of 20 mg·L^−1^) and stirred in a dark room to reach an adsorption–desorption equilibrium. After irradiation at certain time intervals, the concentration of Cr(VI) was analyzed using a UNICO UV-4802 UV-Vis spectrophotometer, and the content of tetracycline hydrochloride was obtained by an Agilent 1100 with a 5-μm, 4.6 × 250 mm Venusil HILIC column and an ultraviolet detector of 356 nm. The intermediate products of Cr(VI) ions and tetracycline hydrochloride were detected by an ICP-OES (Varian 710-ES) and a UPLC-MS system (Waters UPLC Acquity, Quattro Premier XE), respectively. The effects of carbon content, Ag content, solution concentration, pH value, and inorganic ions on the photocatalytic activity of CeO_2_-based composites were investigated using the above process. The photocatalytic durability of the obtained samples was also obtained under the same conditions.

## 3. Results

**Figure 1:**
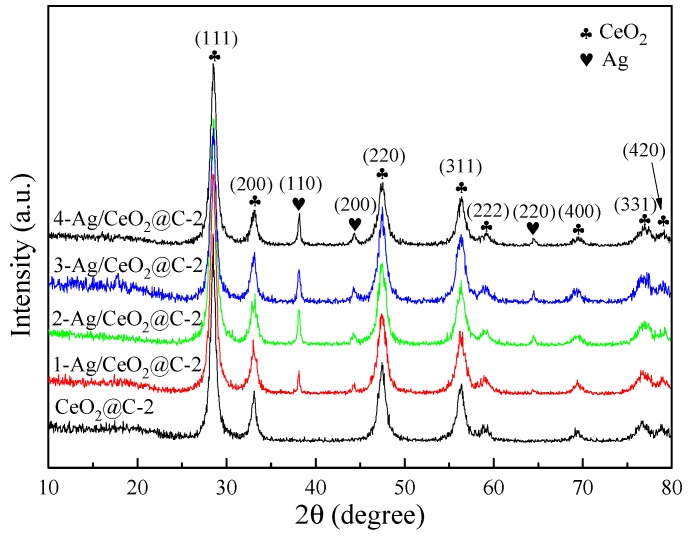
XRD patterns of CeO_2_@C-2 and Ag/CeO_2_@C-2.

The typical peaks of CeO_2_ phases appeared at 28.57°, 33.08°, 47.47°, 56.42°, 59.18°, 69.42°, 76.95°, and 79.15° in XRD patterns of CeO_2_, CeO_2_@C, and Ag/CeO_2_@C (Figure S1 and Figure 1). These peaks were assigned to the (111), (200), (220), (311), (222), (400), (311), and (420) facets of cubic CeO_2_ phases (JCPDS No. 34-0394), respectively [20,23,25]. The peaks of Ag/CeO_2_@C at 38.10°, 44.32°, and 64.49° were ascribed to the (110), (200), and (220) planes of face-centered cubic Ag phases (JCPDS No. 04-0783) [22,23,41]. The diffraction peaks of carbon phases were not detected in the XRD patterns of CeO_2_@C and Ag/CeO_2_@C because they had less carbon content (<5%) and weak amorphous carbon intensity [6,53,54]. With an increase in Ag content, the diffraction peak intensities of Ag (110) and CeO_2_ (111) (Figure 1) respectively increased at around 38.10° and 28.57°, indicating the high crystallinity of Ag/CeO_2_@C.

**Figure 2:**
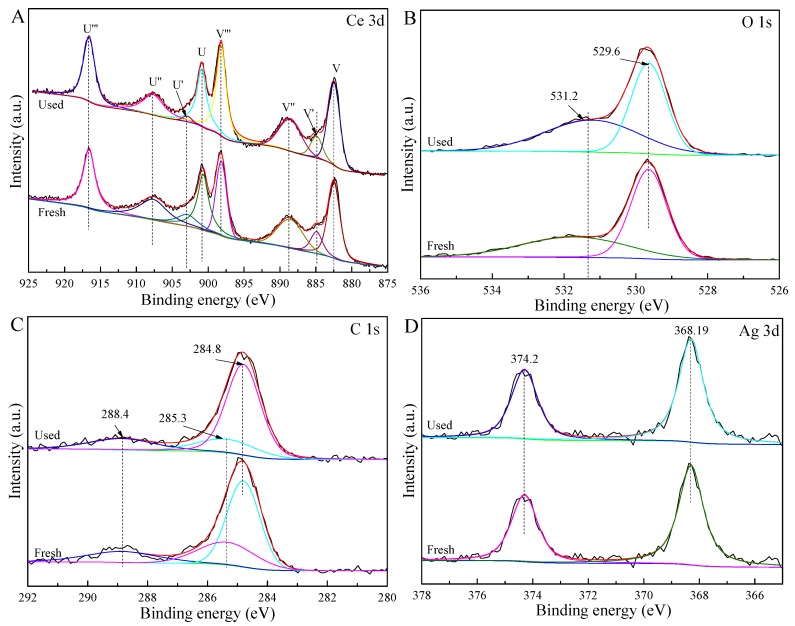
Ce 3d (**A**), O 1s (**B**), C 1s (**C**), and Ag 3d (**D**) X-ray photoelectron spectroscopy (XPS) spectra of fresh and used 3-Ag/CeO_2_@C-2.

XPS was applied to investigate the surface compositions and chemical states of the obtained CeO_2_ composites. The Ce 3d spectrum (Figure 2A, Figures S2A and S3A) was split into eight Gaussian peaks. The peaks at 900.7 eV (U) and 882.5 eV (V) were respectively assigned to Ce 3d_3/2_ and Ce 3d_5/2_, indicating the formation of Ce^3+^ and Ce^4+^ [20,21]. The peaks at 902.9 eV (U′) and 884.9 eV (V′) were ascribed to Ce^3+^ [23]. The peaks at 916.6 eV (U‴), 907.7 eV (U″), 898.2 eV (V‴), and 888.6 eV (V″) were indexed to Ce^4+^ [20]. The relative content of Ce^3+^ could be calculated by the following equation (listed in Table S1):

Ce^3+^ = Ce^3+^/(Ce^3+^ + Ce^4+^) = area (U′ and V′)/Total area. (1)

**Figure 3:**
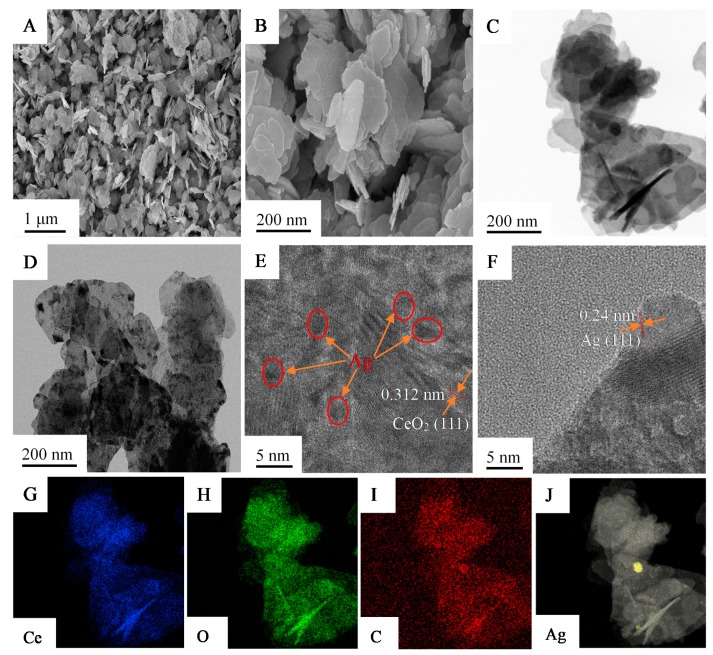
SEM images (**A**,**B**), TEM images (**C**,**D**), high-resolution TEM (HRTEM) images (**E**,**F**), and elemental mapping images (**G**–**J**) of 3-Ag/CeO_2_@C-2.

The surface atomic compositions of these samples were nonstoichiometric values compared to the theoretical values due to the presence of a Ce^3+^ state (Table S1). The Ce^3+^ concentrations of CeO_2_, CeO_2_@C-2, and 3-Ag/CeO_2_@C-2 were, respectively, 12.15%, 14.45%, and 16.54%. The charge compensation may have been responsible for the increase in Ce^3+^ content of CeO_2_@C-2 and 3-Ag/CeO_2_@C-2. It was noticed that the Ce^3+^ content (5.81%) of used 3-Ag/CeO_2_@C-2 after five cycles was lower than that of a fresh sample. This was attributed to the reduced charge compensation under long-term irradiation, leading to inferior photocatalytic stability [15]. The divided Gaussian peaks at 531.2 eV and 529.6 eV (Figure 2B, Figures S2B and S3B ) were attributed to the defect oxygen (or oxygen vacancy) and lattice oxygen, respectively [15,20,31]. The varied ratios of defect oxygen (or oxygen vacancy) to lattice oxygen were greatly related to the Ce^3+^ content in the as-obtained samples. The concentration of the defect oxygen (or oxygen vacancy) in the O 1s XPS spectrum of the used 3-Ag/CeO_2_@C-2 (Figure 2B) was higher than that of a fresh sample. This was due to the released and adsorbed oxygen of CeO_2_ and the defect structure of Ce^3+^–O_v_–Ce^4+^ (O_v_- oxygen vacancy) under reduction and oxidation conditions [38,42]. The C1 spectra of CeO_2_@C-2 (Figure 3C) and fresh and used 3-Ag/CeO_2_@C-2 (Figure 2C) were divided into three Gaussian peaks at 288.4 eV, 285.3 eV, and 284.8 eV, which respectively belonged to C=O, C–OH, and C–C/C=C bonds. The splitting peaks at 374.2 eV and 368.19 eV in the Ag 3d XPS spectrum of fresh and used 3-Ag/CeO_2_@C-2 (Figure 2D) were assigned to Ag 3d_3/2_ and Ag 3d_5/2_, respectively. Although there was no change in the microstructure of used 3-Ag/CeO_2_@C-2 (Figure S4), long-term irradiation induced a difference in the C 1s and Ag 3d XPS spectra between the fresh and used samples (Figure 3C,D), leading to inferior charge transfer and separation [45,47].

The detailed microstructure and surface morphology of CeO_2_, CeO_2_@C, and Ag/CeO_2_@C were obtained by SEM and TEM. All of these samples were irregular nanosheets with thicknesses ranging from 10 nm to 20 nm (Figures S4 and S5, and Figure 3A,B). Compared to the CeO_2_ precursor (Figure S5A,B), there were many more fragments detected in the CeO_2_ after it was treated at high temperature (Figure S5C,D). With the assistance of carbon coating, fewer fragments and agglomerated nanosheets were detected in CeO_2_@C (Figure S5E–G) and Ag/CeO_2_@C (Figure S6), which was further confirmed by the TEM images (Figure S7). With an increase in carbon content, the agglomeration of CeO_2_@C gradually disappeared and even formed single nanosheets, while the specific surface area of CeO_2_@C decreased (Table S2). Although the nanosheet structure of Ag/CeO_2_@C was not affected by the Ag doping, the fragment content increased with increasing Ag-doped content due to the damage effect of NaBH_4_ during the in situ reduction process (Figure S6). In addition, the specific surface area of Ag-doped CeO2@C-2 deceased with increasing Ag-doped content (Table S2). As is shown in Figure 3, 3-Ag/CeO_2_@C-2 had irregular and fragmented nanosheets (Figure 3A,B), and ultrafine Ag nanoparticles with a diameter of around 3 nm (dark section in the red circle) were anchored on the CeO_2_@C-2 (Figure 3C,D and Figure S7E,F), which was evidenced by the HRTEM images (Figure 3E,F). The spacing distances between neighboring lattice fringes of the (111) plane of cubic CeO_2_ and the (111) facet of cubic Ag (Figure 3E,F) were, respectively, 0.312 nm and 0.24 nm, which agreed with the XRD pattern of 3-Ag/CeO_2_@C-2. The elemental distribution of 3-Ag/CeO_2_@C-2 was further obtained by the elemental mapping images. As is shown in Figure 3G–J, there were Ce, O, C, and Ag elements existing in the obtained sample and discontinuous and monodisperse distributions of Ag elements on the bulk surface, indicating the formation of Ag QDs anchored in CeO_2_@C nanosheets.

**Figure 4:**
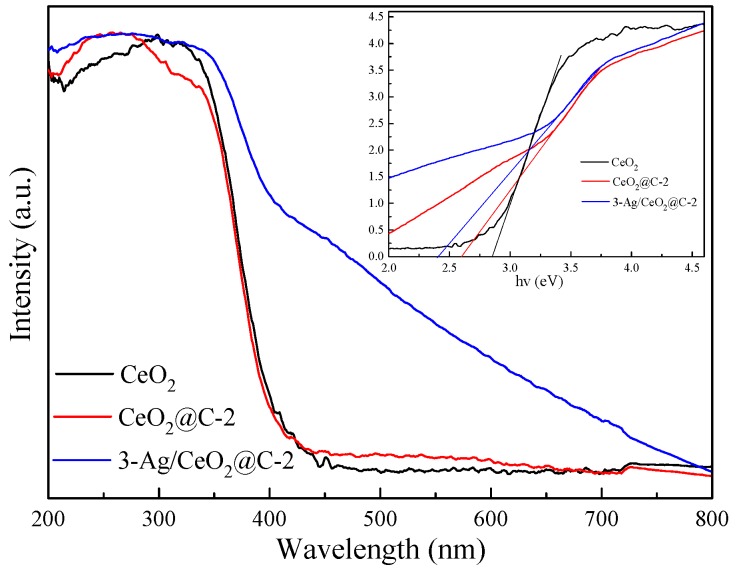
UV–Vis spectra of CeO_2_, CeO_2_@C-2, and 3-Ag/CeO_2_@C-2.

**Figure 5:**
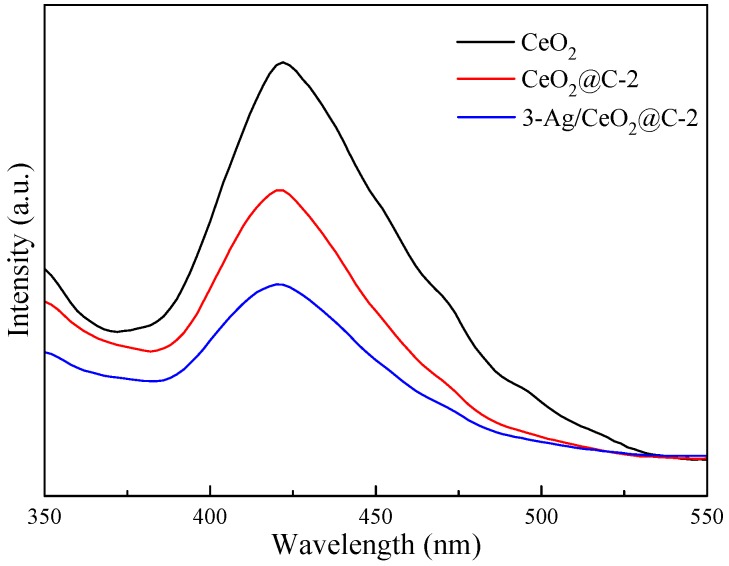
Photoluminescence (PL) spectra of CeO_2_, CeO_2_@C-2, and 3-Ag/CeO_2_@C-2.

The molecular structures of the obtained CeO_2_, CeO_2_@C, and Ag/CeO_2_@C were obtained by FT-IR, as shown in Figures S8 and S9. The peaks at 3440 and 1640 cm^−1^ were ascribed to the stretching vibration and bending vibration of the O–H group of absorbed water and surface hydroxyl [9,11]. The peak at 1539 cm^−1^ was attributed to the H–O–H bending vibration of water molecules. The band peaks around 2921 cm^−1^, 2847 cm^−1^, and 1377 cm^−1^ were due to the bending vibration of the C–H group. The peaks at 2362 cm^−1^ and 2340 cm^−1^ could be assigned to the stretching vibrations of C=O groups of adsorbed CO_2_ in the air. The peaks at 676 cm^−1^, 567 cm^−1^, and 475 cm^−1^ were attributed to the vibration of metal oxygen bonds [41]. The optical properties of CeO_2_, CeO_2_@C-2, and 3-Ag/CeO_2_@C-2 were obtained by UV-Vis DRS, as shown in Figure 4. Compared to CeO_2_ and CeO_2_@C-2, 3-Ag/CeO_2_@C-2 exhibited strong visible-light-harvesting capacity due to the SPR effect of Ag QDs [18,22,23]. According to the plot of (*αhv*)^1/2^ versus (*hv*), the calculated band gap energy of 3-Ag/CeO_2_@C-2 (2.47 eV) was lower than those of CeO_2_ (2.61 eV) and CeO_2_@C-2 (2.86 eV). The photoluminescence (PL) spectra (Figure 5) indicated that the PL peak intensity of 3-Ag/CeO_2_@C-2 was also weaker than those of CeO_2_ and CeO_2_@C-2 after an excitation at a 300-nm wavelength [25]. On the basis of the standard quantum efficiency of 100% formed from the absorbance at the excitation wavelength and the photoluminescence intensity, the estimated fluorescence efficiency of 3-Ag/CeO_2_@C-2 (13.21%) was higher than those of CeO_2_ (5.72%) and CeO_2_@C-2 (8.36%), meaning a lower recombination of charge carriers over 3-Ag/CeO_2_@C-2. The broad emission band around 350–550 nm was responsible for Ce^3+^ ions and oxide defects in CeO_2_. In addition, the combined effect of carbon coating and Ag QD-doping was helpful for efficient charge transfer and high resistance to the recombination of electron–hole pairs [41,45].

**Figure 6:**
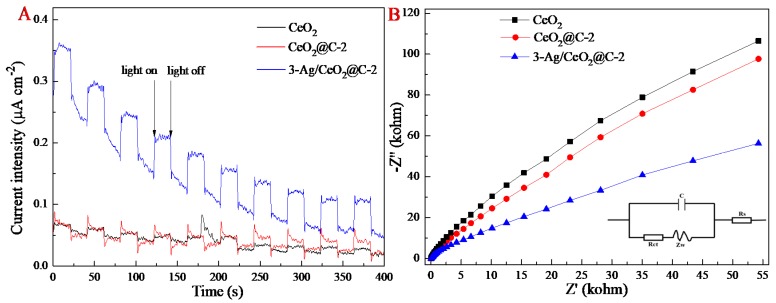
Photocurrent–time response (**A**) and electrochemical impedance spectroscopy (EIS) Nyquist plots (**B**) of 3-Ag/CeO_2_@C-2.

The separation efficiency of photocatalytic electron–hole pairs was evaluated by electrochemical impedance spectroscopy (EIS), in which the arc radius represented the transfer rate of the photocatalyst charge. In contrast to CeO_2_ and CeO_2_@C-2, 3-Ag/CeO_2_@C-2 exhibited a higher transient photocurrent under visible-light irradiation (Figure 6A) and a smaller arc radius of electrochemical impedance (Figure 6B). The small radius of 3-Ag/CeO_2_@C-2 suggested that the low resistance was suitable for the efficient separation of charge carriers in the obtained 3-Ag/CeO_2_@C-2 photocatalyst. Although a large specific surface area is favorable in reducing the diffusion length of charge carriers and further prompting charge transfer, the SPR effect of Ag QDs and a tight interface between carbon and CeO_2_ nanosheets are crucial for the enhanced separation of charge carriers and the restrained recombination of photoexcited electron–hole pairs [47,48]. Mott–Schottky curves of the obtained CeO_2_, CeO_2_@C-2, and 3-Ag/CeO_2_@C-2 were performed for an evaluation of the semiconductor type and flat band potentials (*V_fb_*), where the *V_fb_* values of these samples could be obtained from the *x* intercept by prolonging the linear part of the Mott–Schottky curves on the potential axis (Figure S10A). The *V_fb_* values of CeO_2_, CeO_2_@C-2, and 3-Ag/CeO_2_@C-2 were −0.83 V, −0.65 V, and −0.58 V versus a KCl-saturated calomel electrode, respectively. Hence, the valence band values of CeO_2_, CeO_2_@C-2, and 3-Ag/CeO_2_@C-2 were −0.59 V, −0.41 V, and −0.34 V, respectively. According to the calculated band gap energy, the conduction band values of CeO_2_, CeO_2_@C-2, and 3-Ag/CeO_2_@C-2 were 2.27 V, 2.20 V, and 2.13 V, respectively. Compared to 3-Ag/CeO_2_@C-2 alone with a bias voltage of 1.0 V, the addition of visible-light irradiation with a light-power intensity of 240 mW cm^−2^ (λ > 420 nm) could enhance the current response (Figure S10B), indicating efficient photoinduced charge in the visible light region.

**Figure 7:**
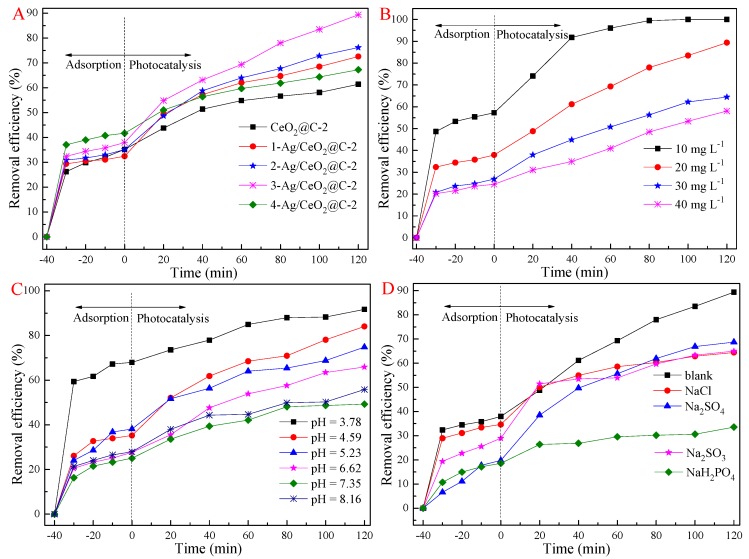
Effect of Ag content on the photocatalytic activity of CeO_2_@C-2 (**A**) and the effects of Cr(VI) concentration (**B**), pH value (**C**), and inorganic ions (**D**) on the photocatalytic activity of 3-Ag/CeO_2_@C-2.

CeO_2_-based composites were performed for the visible-light-driven photocatalytic reduction of Cr(VI) ions and the photodegradation of tetracycline hydrochloride (TCH). Compared to CeO_2_ nanosheets, carbon-coated composites exhibited better adsorption–photocatalytic activity under the same conditions, especially CeO_2_@C-2 (Figure S11). The enhanced adsorption–photocatalysis behaviors of CeO_2_@C were due to sufficient active sites of carbon shells and the oxygen vacancy of CeO_2_ cores. In addition, this was ascribed to the trap effect of carbon shells for enhanced charge transfer and efficient charge carrier separation [9,49,50]. With the assistance of carbon shells, the excited electrons could efficiently escape from the conduction band (CB) of CeO_2_ to amorphous carbon, achieving the separation of electron–hole pairs and restraining their recombination. The SPR effect of Ag QDs could further strengthen the photocatalytic activity of CeO_2_@C-2 (Figure 7A). The photocatalytic activity of Ag/CeO_2_@C-2 for Cr(VI) removal increased and then decreased with an increase in Ag-doping content. In addition, Ag/CeO_2_@C-2 composites also exhibited excellent photocatalytic activity for the visible-light-driven photodegradation of TCH compared to CeO_2_@C-2, as shown in Figure S12. A similar tendency in the effects of Ag-doping content on photocatalytic activity was obtained for the removal of TCH in the visible-light region. Among these Ag QDs doped composites, CeO_2_@C-2 and 3-Ag/CeO_2_@C-2 with an Ag-doping content of 5.41% presented the best photocatalytic activity. Due to the limited active sites, an excess of Cr(VI) ions could not efficiently access the active sites, leading to inferior photocatalytic efficiency. Hence, the removal efficiency of 3-Ag/CeO_2_@C-2 decreased with increasing concentrations of Cr(VI) ions (ranging from 10 mg L^−1^ to 40 mg·L^−1^ (Figure 7B)). The optimum pH value facilitated the reaction between hydroxyl (OH^−^) (or H^+^) ions and radical species (such as e^−^ and h^+^) to generate ·O_2_^−^ and ·OH radicals [20,47]. In a photocatalytic reaction system, H^+^ ions can react with ·O_2_^−^ to form ·OOH radicals, and ·OOH can react with H^+^ ions to generate H_2_O_2_ [3]. Subsequently, H_2_O_2_ is likely to react with e^−^ to form ·OH and OH^−^, of which OH^−^ ions are scavenged by h^+^ to produce ·OH [9]. In addition, ·OH is also generated from the reaction between H_2_O and h^+^ [15]. An excess of OH^−^ ions can quench the above chain reactions, leading to inferior photocatalytic activity (Figure 7C).

**Figure 8:**
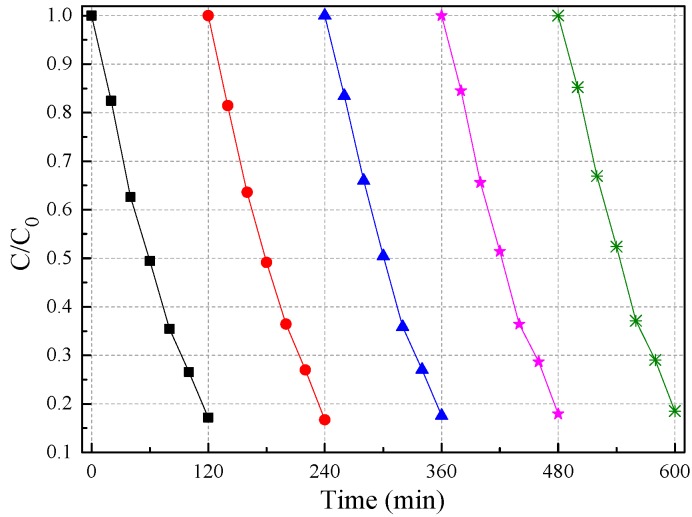
Photocatalytic stability of 3-Ag/CeO_2_@C-2 in Cr(VI) removal in the visible-light region.

Inorganic ions such as chlorine (Cl^−^), sulfite (SO_3_^2−^), sulfate (SO_4_^2−^), and phosphate (H_2_PO_4_^−^) could affect the photocatalytic activity of 3-Ag/CeO_2_@C-2 in Cr(VI) removal. As is shown in Figure 7D, inorganic ions could restrain photocatalytic activity compared to 3-Ag/CeO_2_@C-2 alone in Cr(VI) removal under the same conditions. The removal efficiency of Cr(VI) was remarkably inhibited by H_2_PO_4_^−^ in comparison to Cl^−^, SO_4_^2−^, and SO_3_^2−^. The impeding effect of inorganic ions was due to ·OH scavengers and reduced active sites [9,10]. On the one hand, inorganic ions served as ·OH scavengers and competed with Cr(VI) ions for ·OH radicals. Although the ion radicals were suitable for the oxidizing pollutants, their low oxidoreductive potential induced inferior photocatalytic rates compared to ·OH, especially in acidic conditions [13]. On the other hand, inorganic ions could adsorb on the surface of 3-Ag/CeO_2_@C-2, leading to decreased surface-active sites for Cr(VI) ions. As is shown in Figure 8, the photocatalytic activity of 3-Ag/CeO_2_@C-2 remained slightly changed after five cycles. However, the mass loss of 3-Ag/CeO_2_@C-2 in the sedimentation and transferring processes induced a decrease in photocatalytic activity in each cycle test. SEM images (Figure S4) confirmed that the structure of the used 3-Ag/CeO_2_@C-2 remained unchanged after five cycles of photocatalytic reactions. However, the surface compositions and chemical states of the used 3-Ag/CeO_2_@C-2 (Figure 2) were different from the fresh composites due to long-term photocorrosion.

**Figure 9:**
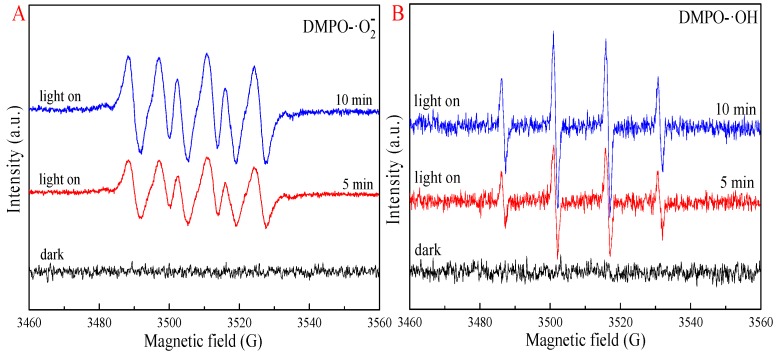
Electron spin resonance (ESR) spectra of 3-Ag/CeO_2_@C-2 for DMPO–·O_2_^−^ in methanol (**A**) and DMPO–·OH in aqueous (**B**).

**Figure 10:**
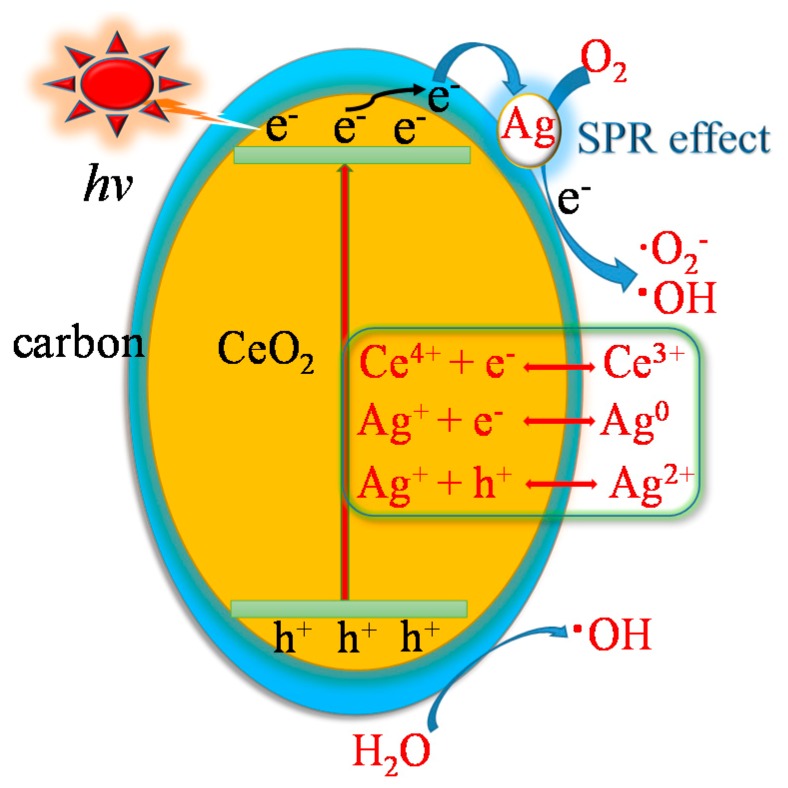
Photocatalytic mechanism of 3-Ag/CeO_2_@C-2.

As is shown in Figure 9, ESR signals of photoinduced radicals such as ·OH and ·O_2_^−^ were clearly obtained, in which the intensities increased with an increase in the irradiation time of visible light. Quenching testing (Figure S13) indicated that the photocatalytic reaction was suppressed by tert-butyl alcohol (*t*-BuOH), benzoquinone (BQ), and ethylenediaminetetraacetic acid disodium salt (EDTA-2Na), especially *p*-BQ and *t*-BuOH. It was concluded that the ·OH and ·O_2_^−^ radicals were vital for the photocatalytic activity of 3-Ag/CeO_2_@C-2. The enhanced photocatalytic capacity was due to the SPR effect of Ag QDs, an electron trapper of carbon shells, and the redox activity of the Ce(III)/Ce(IV) coupling. In other words, the photocatalytic activity was related to the amount of Ag^+^ serving as an electron acceptor (Ag^2+^ ↔ Ag^0^) and/or a hole donor (Ag^2+^ ↔ Ag ^+^) and the concentration of Ce^3+^ and oxygen vacancies, which could promote the localization of charge carriers and prolong the separation of electron–hole pairs via trapping at energy levels close to the valance band or conduction band [13]. Previous work has suggested that the temperature-programmed reduction (TPR) of peaks belonging to the conversion of Ag^2+^ and Ag^+^ into Ag^0^ is obtained at 405 K and 421 K, respectively [55]. Hence, a reaction between e^−^/h^+^ pairs and Ag^0^/Ag^+^/Ag^2+^ ions is likely to promote the photoreduction of Cr(VI) ions. The possible photocatalytic mechanism of the 3-Ag/CeO_2_@C-2 heterojunction is proposed in Figure 10. Under visible-light irradiation, the photoexcited electrons from the valance band (O_2p_, VB = −0.56 eV) to the conduction band (Ce_4f_, CB = 2.30 eV) of CeO_2_ were trapped by carbon shells and then transferred to Ag QDs (Equation (1)). The SPR effect of Ag QDs could strengthen the amount of photoexcited electron–hole pairs. The photogenerated electrons were scavenged by O_2_ molecules (E_O2/O_·_2_^−^ = −0.33 eV/NHE, normal hydrogen electrode) in the atmosphere and in the water solution to yield ·O_2_^−^ and •OH radicals (Equations (3)–(5)): meanwhile, h^+^ and ·O_2_^−^ could react with H_2_O molecules to form an ·OH radical (E_H2O, OH_^−^_/•OH_ = 1.99 eV/NHE) (Equations (6) and (7)) [56,57]. In addition, OH− ions were adsorbed on the h^+^ of the valance band to generate an ·OH radical. These radicals were responsible for the efficient photocatalytic reduction of Cr(VI) into Cr(III) ions (Equations (8)–(13)), which was confirmed by the Cr 2p XPS spectrum of the used 3-Ag/CeO_2_@C-2 after five cycles of photocatalytic reaction (Figure S14). The splitting peaks at 588.2 eV and 579.2 eV were indexed to Cr(VI), and the peaks at 586.2 eV and 576.4 eV were indexed to Cr(III), indicating a conversion of Cr(VI) ions into Cr(III) ions according to the electron transfer process [56]. Under acidic conditions, Cr(VI) ions could react with electrons and ·OH to generate Cr(III) ions (Equations (8)–(11)). Under alkaline conditions, low H^+^ ions could react with Cr(VI) ions to form Cr^3+^ ions (Equation (12)) and further generate Cr(OH)_3_ loaded on the surface of 3-Ag/CeO_2_@C-2 bulks (Equation (13)), leading to inferior light adsorption capacity [58,59,60]. In addition, TCH could react with ·OH and h^+^ to form small molecules, which was evaluated by UPLC–MS (Figure S15). Under visible-light irradiation, TCH molecules were degraded via N–C bond cleavage and hydroxylation, and then these intermediates could react with the ·OH radical to destroy C2–C3 double bonds and eliminate NH_3_: they subsequently disintegrated into small molecules and even H_2_O and CO_2_ through demethylation, deamination, and ·OH attack [55,56]. Ag QDs doping and carbon shells could serve as an interfacial charge transfer medium and a recombination center, accelerating the photocatalytic reaction:

(2) $$\mathrm{Ag}/{\mathrm{CeO}}_{2}@C+\mathrm{hv}\to h^{+}+e^{-}$$

(3) $$O_{2}+e^{-}\to \;\cdot O_{2}^{-}$$

(4) $$O_{2}+e^{-}+H^{+}\to \;\cdot \mathrm{OOH}$$

(5) $$\cdot O_{2}^{-}\;+\cdot \mathrm{OOH}+H^{+}\to 2\;\cdot \mathrm{OH}+O_{2}$$

(6) $$H_{2}O+h^{+}\to \;\cdot \mathrm{OH}+H^{+}$$

(7) $$\cdot O_{2}^{-}\;+2H_{2}O+h^{+}\to 4\cdot \mathrm{OH}$$

(8) $$14H^{+}+{\mathrm{Cr}}_{2}O_{7}^{2-}+6e^{-}\to 2{\mathrm{Cr}}^{3+}+7H_{2}O$$

(9) $$7H^{+}+{\mathrm{HCrO}}_{4}^{-}+3e^{-}\to {\mathrm{Cr}}^{3+}+4H_{2}O$$

(10) $$\cdot \mathrm{OH}+{\mathrm{HCrO}}_{4}^{-}+8H^{+}+4e^{-}\to {\mathrm{Cr}}^{3+}+5H_{2}O$$

(11) $$\cdot \mathrm{OH}+{\mathrm{Cr}}_{2}O_{7}^{2-}+15H^{+}+7e^{-}\to 2{\mathrm{Cr}}^{3+}+8H_{2}O$$

(12) $$8H^{+}+{\mathrm{CrO}}_{4}^{2-}+3e^{-}\to {\mathrm{Cr}}^{3+}+4H_{2}O$$

(13) $$4H_{2}O+2{\mathrm{CrO}}_{4}^{2-}+H^{+}\to 2\mathrm{Cr}{\left(\mathrm{OH}\right)}_{3}+3{\mathrm{OH}}^{-}$$

## 4. Conclusions

Ag/CeO_2_@C nanosheets presented better photocatalytic activity than did CeO_2_ and CeO_2_@C for Cr(VI) removal in the visible-light region. The photocatalytic activity of Ag/CeO_2_@C increased and then decreased with an increase in carbon content, Ag doping content, and pH value. Inorganic ions and Cr(VI) content had a negative effect on the removal efficiency of Cr(VI) under the same conditions. The best removal efficiency and the most superior photocatalytic stability after five cycles were achieved by 3-Ag/CeO_2_@C-2 in the visible-light-driven removal of Cr(VI) ions as well as TCH. The SPR effect of Ag QDs, an electron trapper of carbon shells, and the redox activity of the Ce(III)/Ce(IV) coupling played a vital role in the transfer and separation of charge carriers. •O_2_^−^ and •OH radicals were the primary active species of 3-Ag/CeO_2_@C-2 in the photocatalytic system.

## Supplementary Materials

The following are available online at https://www.mdpi.com/2079-4991/9/11/1643/s1: Figure S1: XRD patterns of CeO_2_ and CeO_2_@C; Figure S2: XPS spectra of CeO_2_; Figure S3: XPS spectra of CeO_2_@C-2; Figure S4: SEM images of used 3-Ag/CeO_2_@C-2; Figure S5: SEM images of CeO_2_ precursor (A,B), CeO_2_ (C,D), CeO_2_@C-1 (E,F), CeO_2_@C-2 (G,H), and CeO_2_@C-3 (I,J); Figure S6: SEM images of CeO_2_@C-2 (A,B), 1-Ag/CeO_2_@C-2 (C,D), 2-Ag/CeO_2_@C-2 (E,F), 3-Ag/CeO_2_@C-2 (G,H), and 4-Ag/CeO_2_@C-2 (I,J); Figure S7: TEM images of CeO_2_ (A,B), CeO_2_@C-2 (C,D), and 3-Ag/CeO_2_@C-2 (E,F); Figure S8: FT-IR spectra of CeO_2_ and CeO_2_@C composites; Figure S9: FT-IR spectra of CeO_2_@C-2 and Ag/CeO_2_@C-2 composites; Figure S10: Mott–Schottky curves of CeO_2_, CeO_2_@C-2, and 3-Ag/CeO_2_-2 (A) and U–I curves of 3-Ag/CeO_2_-2 (B); Figure S11: Effect of carbon content on the photocatalytic activity of CeO_2_@C composites in the removal of Cr(VI) in the visible-light region, (Cr(VI) content of 20 mg·L^−1^, catalyst dosage of 40 mg, solution volume of 100 mL, and optical power density of 600 mW·cm^−2^); Figure S12: Effect of Ag content on the photocatalytic activity of Ag/CeO_2_@C-2 composites in the removal of tetracycline hydrochloride in the visible-light region (tetracycline hydrochloride content of 30 mg·L^−1^, catalyst dosage of 40 mg, solution volume of 100 mL, and optical power density of 600 mW cm^−2^); Figure S13: Reactive species trapping experiments over Ag/CeO_2_@C-2; Figure S14: Cr 2p XPS spectrum of used Ag/CeO_2_@C-2 after five cycles; Figure S15: HPLC/MS spectrum of TCH over Ag/CeO_2_@C-2 in visible-light region; Table S1: Atomic ratio and Ce^3+^ ratio of CeO_2_-based samples; Table S2: Texture parameters of CeO_2_-based samples.
