# Supplementary material for: Enhanced Visible-Light Photocatalytic Activity of Ag QDs Anchored on CeO2 Nanosheets with a Carbon Coating
Source: Nanomaterials (Basel). 2019 Nov 19;9(11):1643. doi: 10.3390/nano9111643 (PMC6915373; doi:10.3390/nano9111643)
Supplement: Supplementary file 1 [file nanomaterials-09-01643-s001.pdf]

Supported Information

Enhanced Visible-Light Photocatalytic Activity of Ag QDs Anchored on CeO_2 Nanosheets with a Carbon Coating

Xiaogang Zheng [1,2], Qian Chen [2], Sihao Lv [1,*], Xiaojin Fu [2], Jing Wen [3,*] and Xinhui Liu [4]

1 Research Center for Eco-Environmental Engineering, Dongguan University of Technology, Dongguan 523808, China; zhengxg123456@163.com (X.Z.)

2 College of Chemistry and Chemical Engineering, Neijiang Normal University, Neijiang 641100, China; cq18140267436@163.com (Q.C.); fu-xj2007@163.com (X.F.)

3 Key Laboratory of Comprehensive and Highly Efficient Utilization of Salt Lake Resources, Key Laboratory of Salt Lake Resources Chemistry of Qinghai Province, Qinghai Institute of Salt Lakes, Chinese Academy of Sciences, Xining 810008, China

4 State Key Laboratory of Water Environment Simulation, School of Environment, Beijing Normal University, Beijing 100875, China; xhliu@bnu.edu.cn (X.L.)

* Correspondence: lvsh@dgut.edu.cn (S.L.); wj580420@163.com (J.W.); Tel.: +86-0769-22862965 (S.L.); +86-0971-7762180 (J.W.)

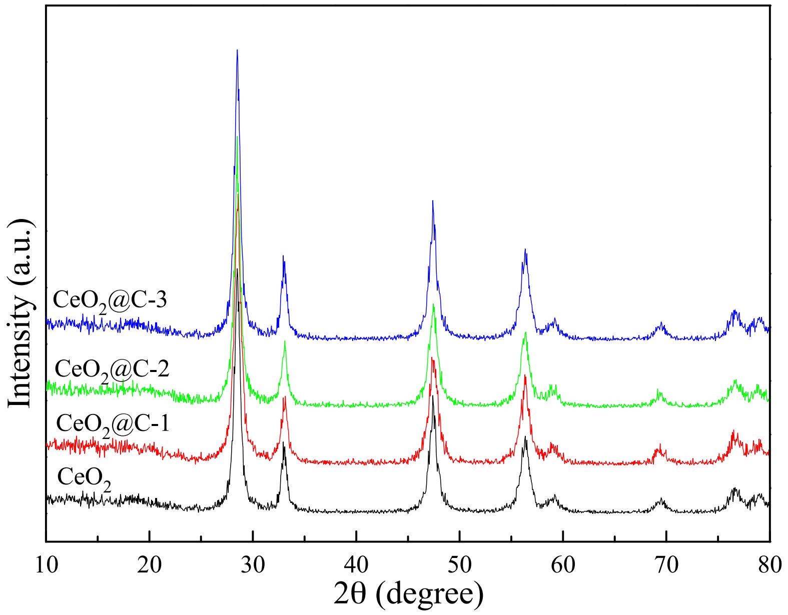

Figure S1. XRD patterns of CeO_2 and CeO_2@C.

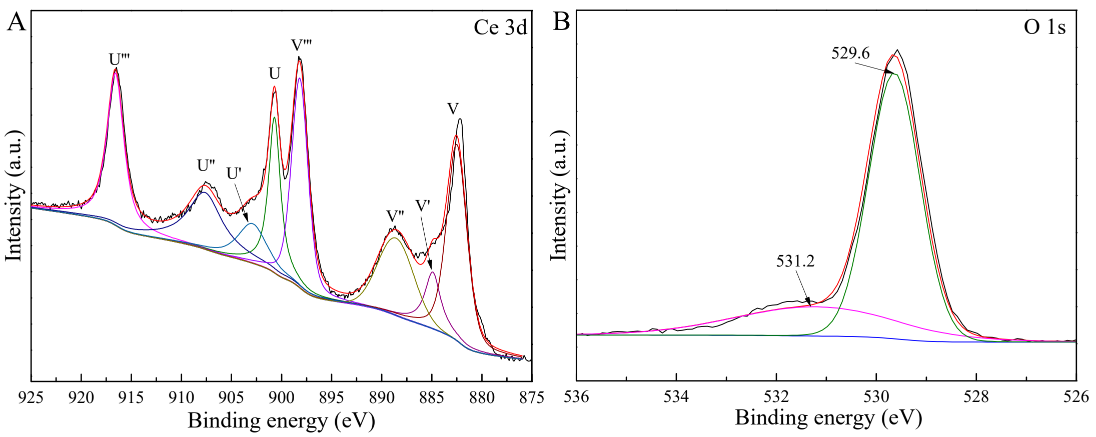

Figure S2. XPS spectra of CeO_2.

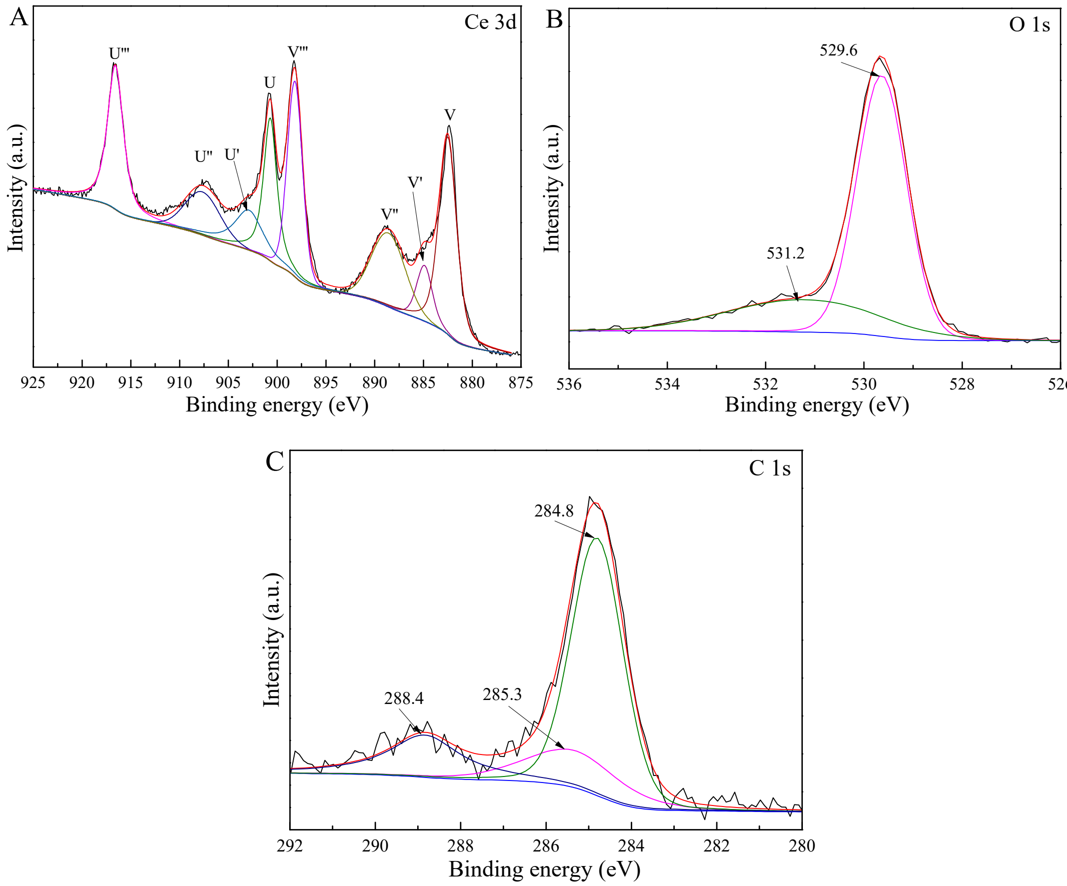

Figure S3. XPS spectra of CeO_2@C-2.

Table S1. Atomic ratio and Ce^{3+} ratio of CeO_2-based samples.

Samples	Atomic ratio (%)				Ce^{3+} ratio (%)
	Ce	O	C	Ag	
CeO_2	27.84	72.16	/	/	12.15
CeO_2@C-2	16.66	45.91	37.28	/	14.45
3-AgCeO_2@C-2	13.14	45.35	40.49	1.02	16.54
Used 3-AgCeO_2@C-2	10.54	47.84	40.37	1.24	5.81

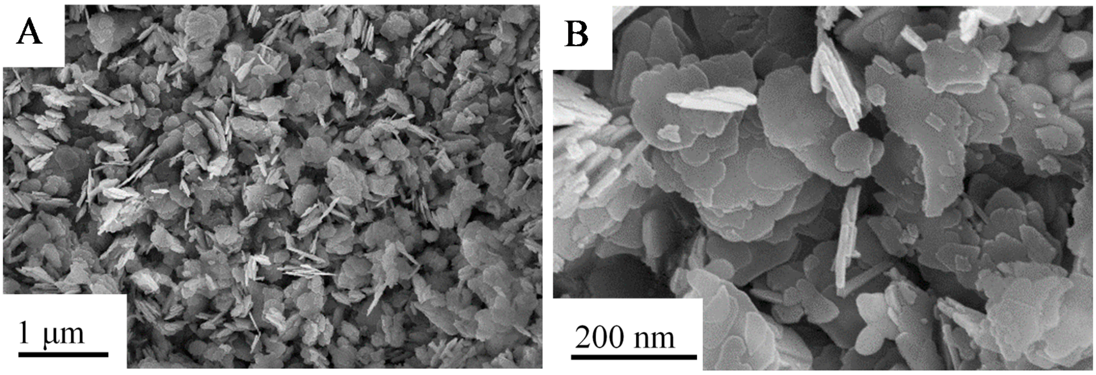

Figure S4. SEM images of used 3-Ag/CeO_2@C-2.

Table S2. Texture parameters of CeO_2-based samples.

Samples	Ag content (%) [a]	Specific surface area (m^2 g^{-1})	Pore size (nm)	Pore volume (cm^3 g^{-1})
CeO_2	/	68.76	3.56	0.096
CeO_2@C-1	/	60.67	3.75	0.083
CeO_2@C-2	/	57.42	3.48	0.074
CeO_2@C-3	/	51.27	3.17	0.081
1-Ag/CeO_2@C-2	3.25	56.15	3.63	0.052
2-Ag/CeO_2@C-2	4.78	49.83	3.44	0.038
3-Ag/CeO_2@C-2	5.41	46.56	3.33	0.039
4-Ag/CeO_2@C-2	6.83	42.74	3.74	0.041

[a] Ag content was measured by ICP-OES.

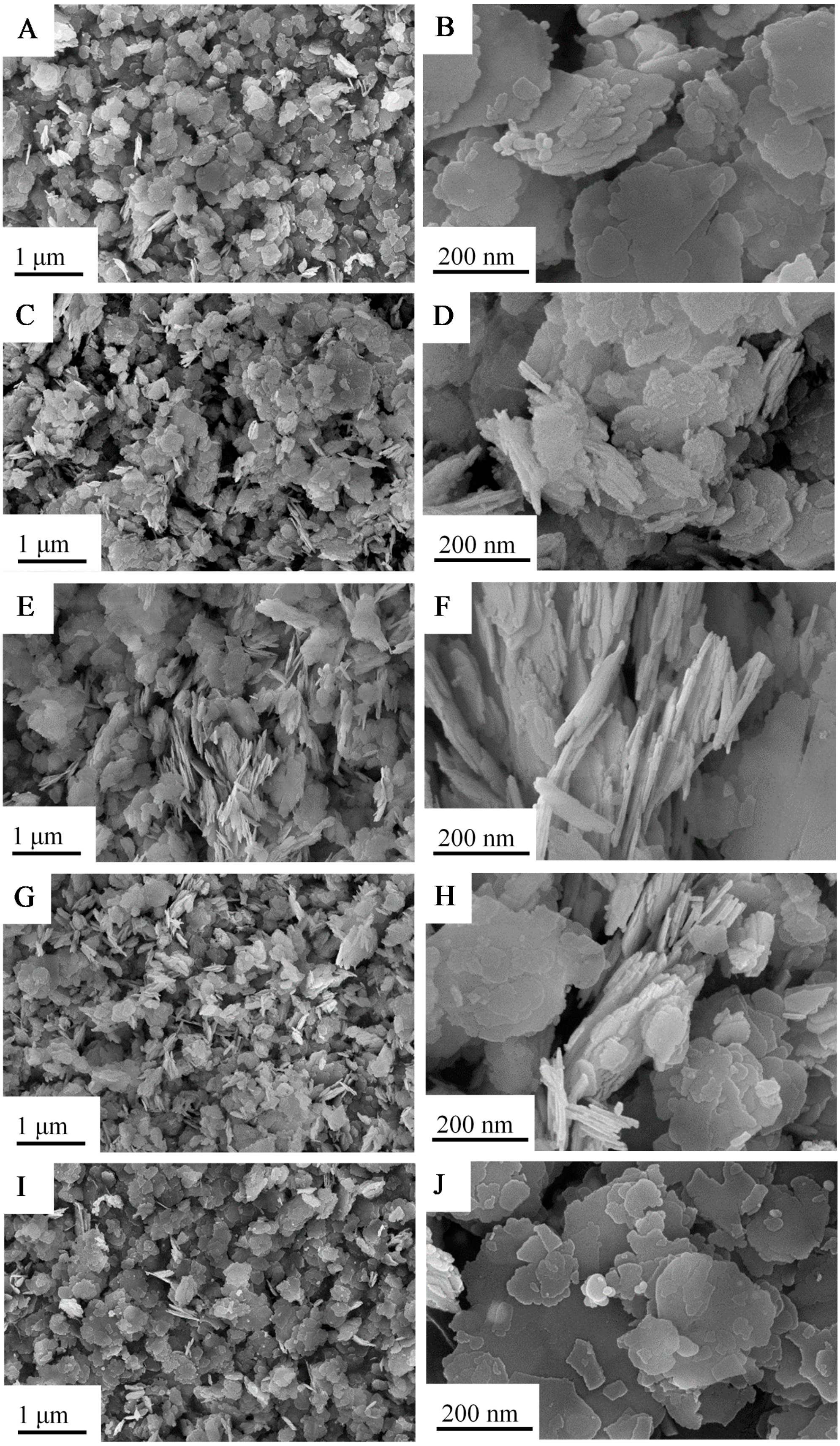

Figure S5. SEM images of CeO_2 precursor (A and B), CeO_2 (C and D), CeO_2@C-1 (E and F), CeO_2@C-2 (G and H) and CeO_2@C-3 (I and J).

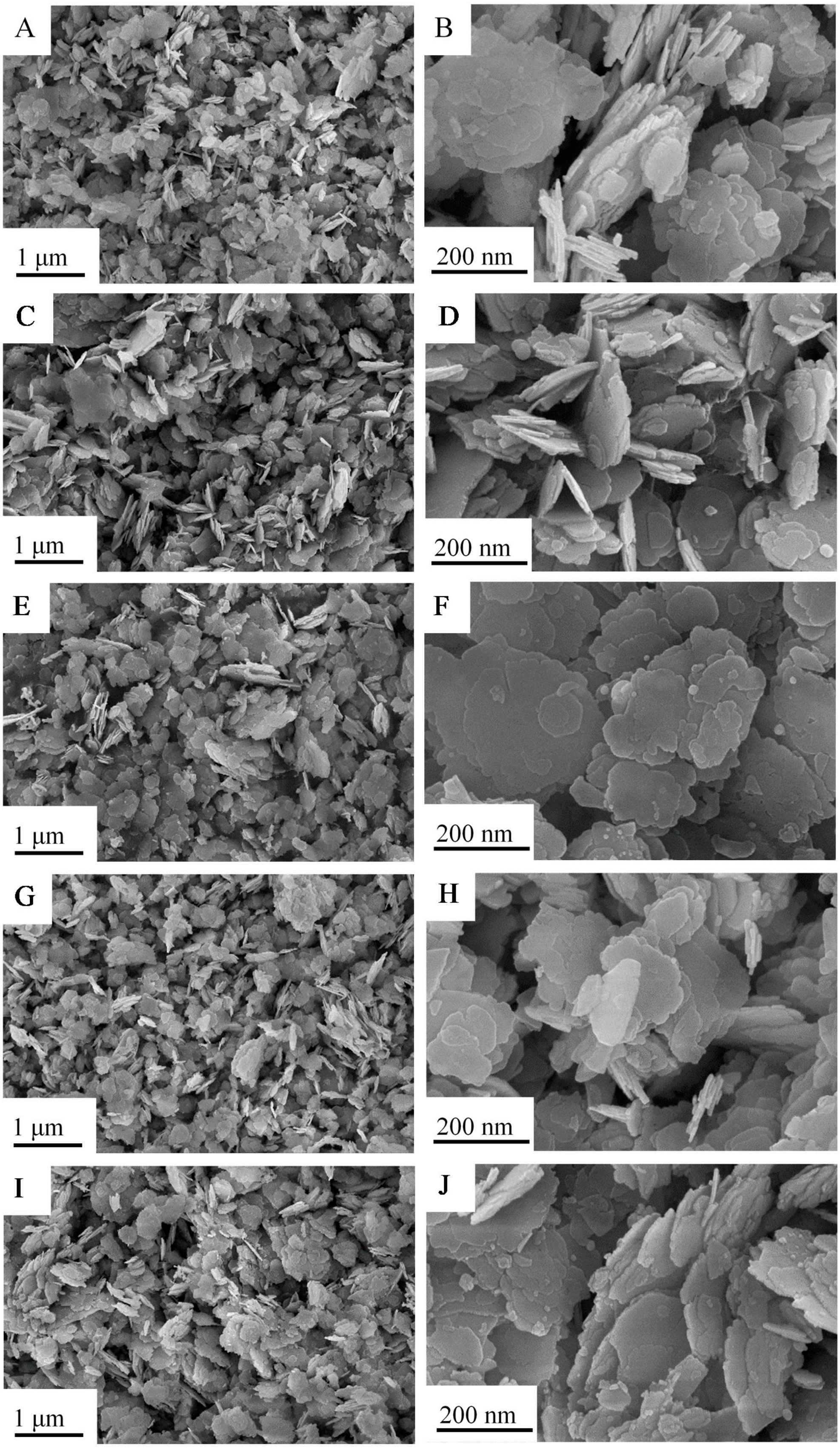

Figure S6. SEM images of CeO_2@C-2 (A and B), 1-Ag/CeO_2@C-2 (C and D), 2-Ag/CeO_2@C-2 (E and F), 3-Ag/CeO_2@C-2 (G and H) and 4-Ag/CeO_2@C-2 (I and J).

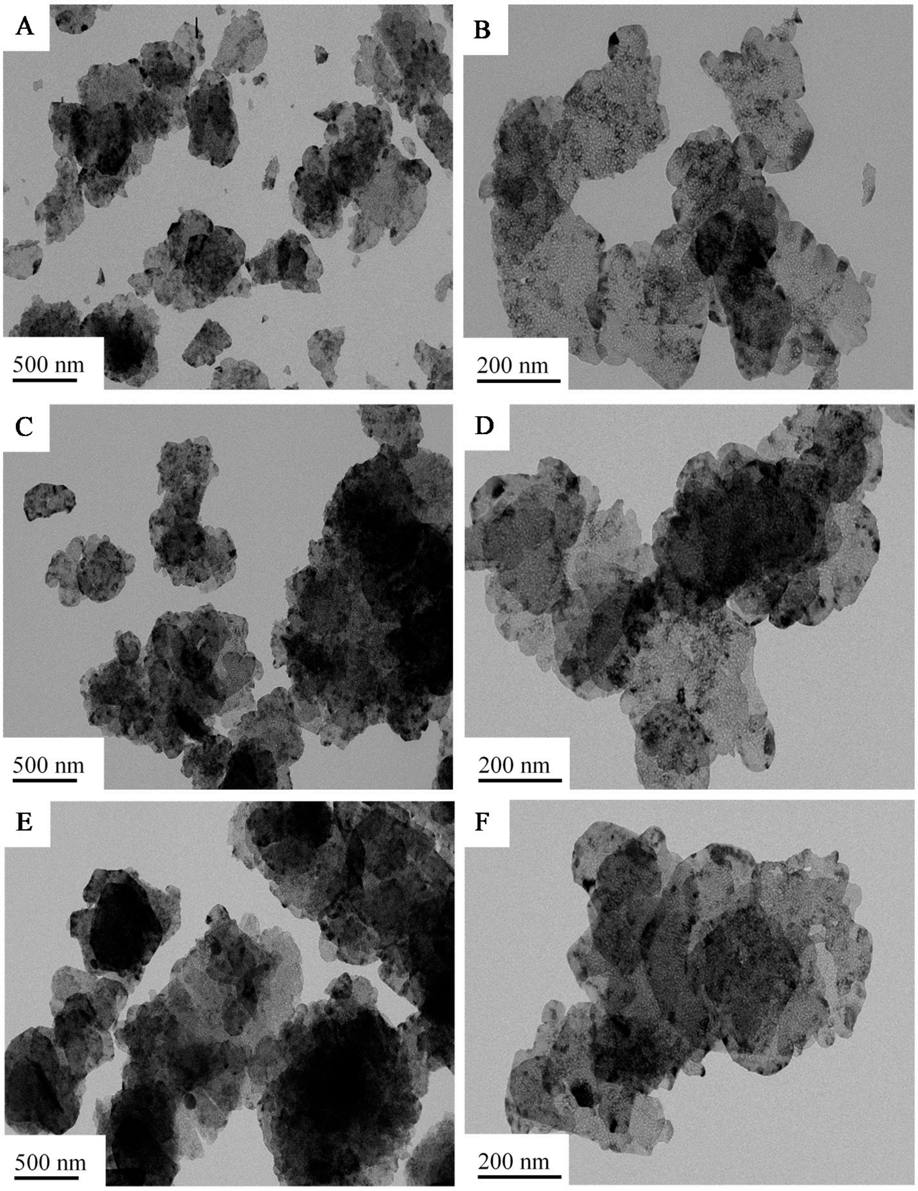

Figure S7. TEM images of CeO_2 (A and B), CeO_2@C-2 (C and D) and 3-Ag/CeO_2@C-2 (E and F).

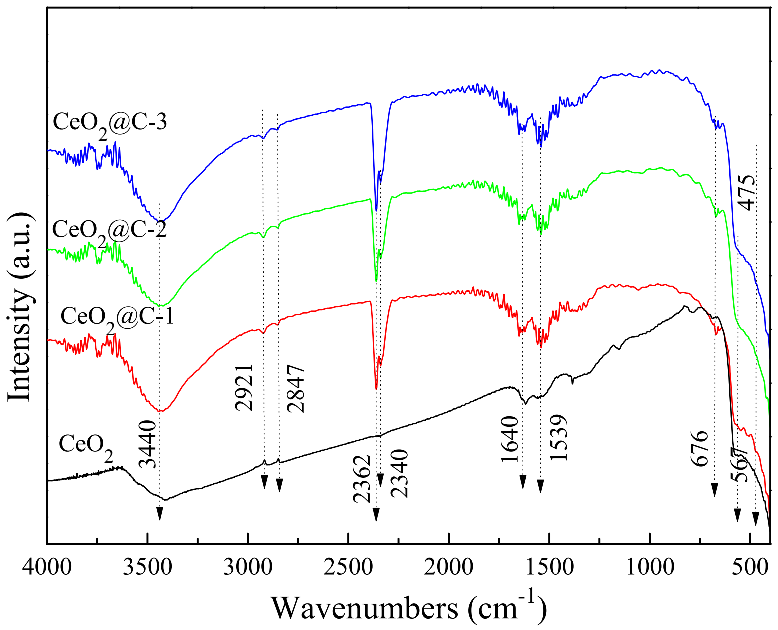

Figure S8. FT-IR spectra of CeO_2 and CeO_2@C composites.

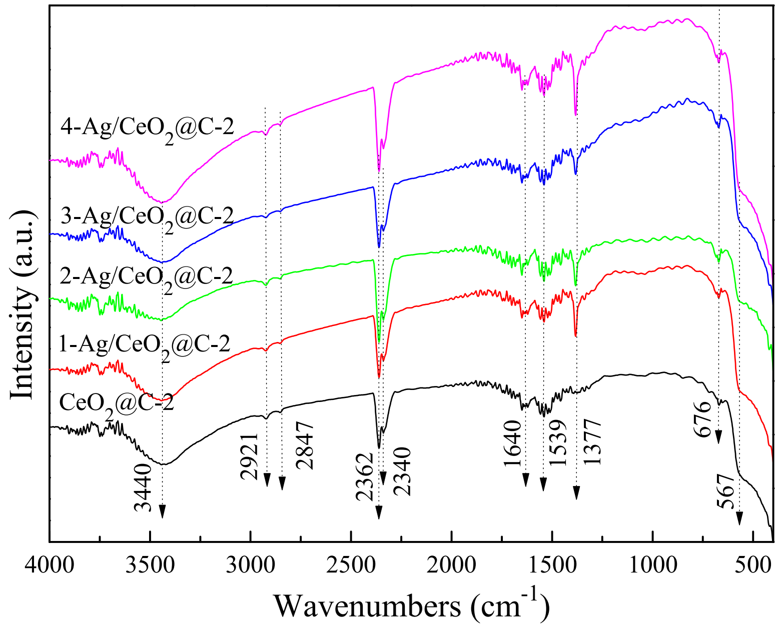

Figure S9. FT-IR spectra of CeO_2@C-2 and Ag/CeO_2@C-2 composites.

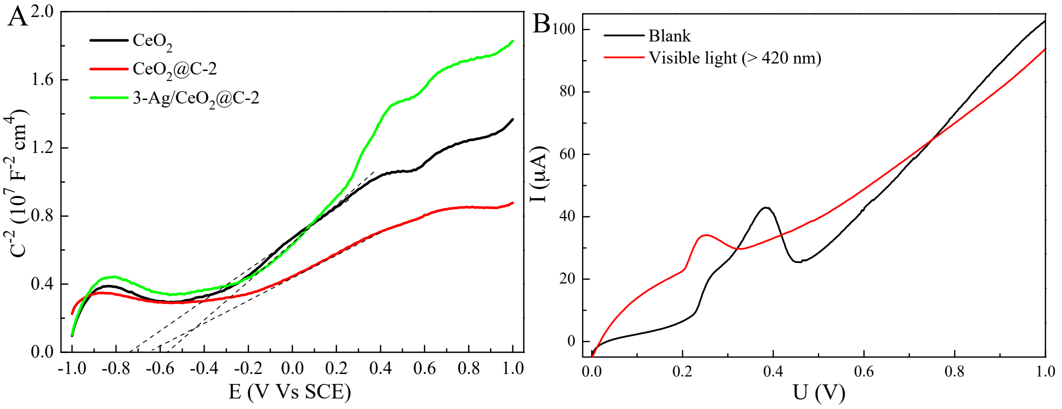

Figure S10. Mott-Schottky curves of CeO_2, CeO_2@C-2 and 3-Ag/CeO_2-2 (A), and U-I curves of 3-Ag/CeO_2-2 (B).

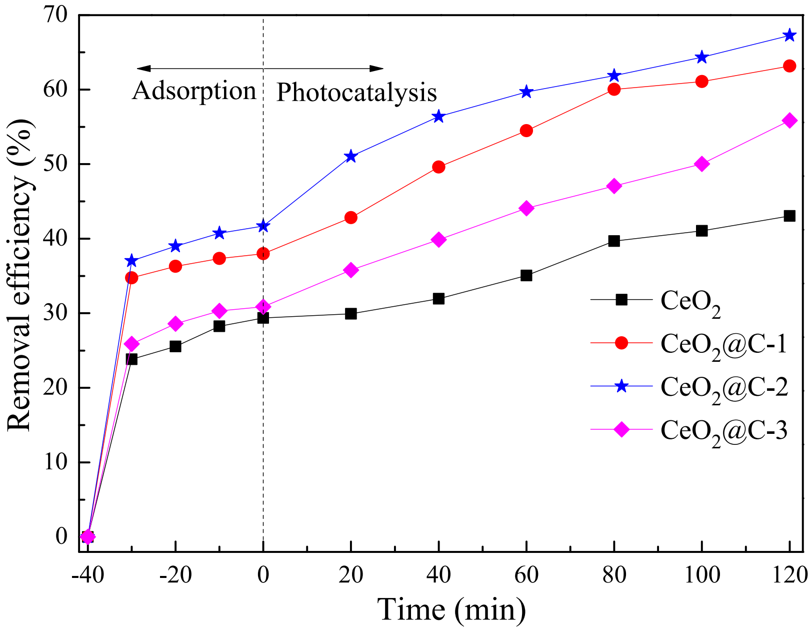

Figure S11. Effect of carbon content on the photocatalytic activity of CeO_2@C composites for removal of Cr(VI) in visible light region. (Cr(VI) content of 20 mg L^{-1}, catalyst dosage of 40 mg, solution volume of 100 mL, optical power density of 600 mW cm^{-2})

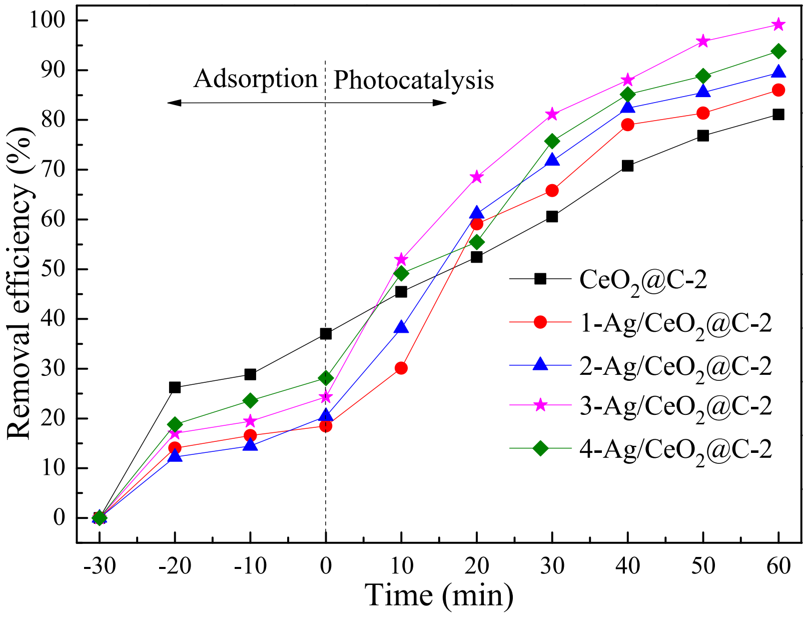

Figure S12. Effect of Ag content on the photocatalytic activity of Ag/CeO_2@C-2 composites for removal of tetracycline hydrochloride in visible light region. (Tetracycline hydrochloride content of 30 mg L^{-1}, catalyst dosage of 40 mg, solution volume of 100 mL, optical power density of 600 mW cm^{-2})

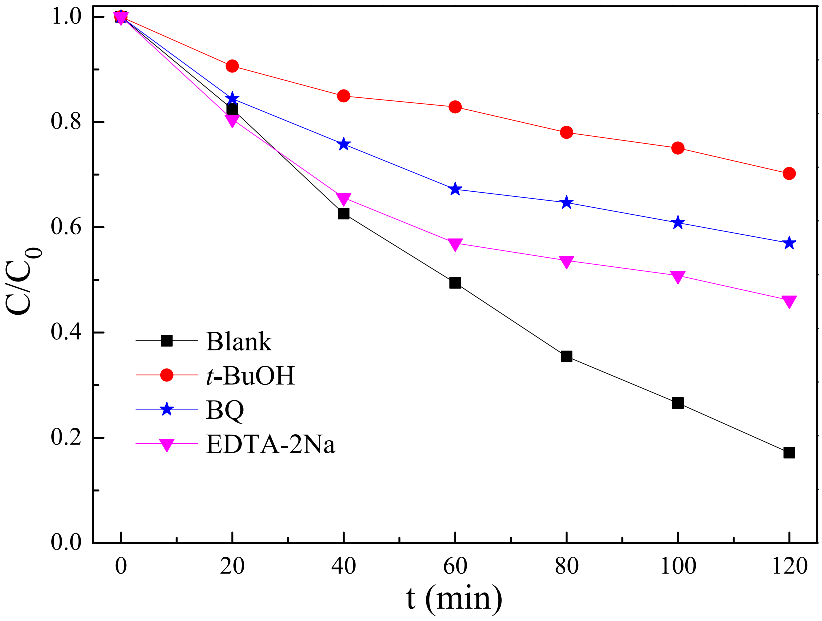

Figure S13. Reactive species trapping experiments over Ag/CeO_2@C-2.

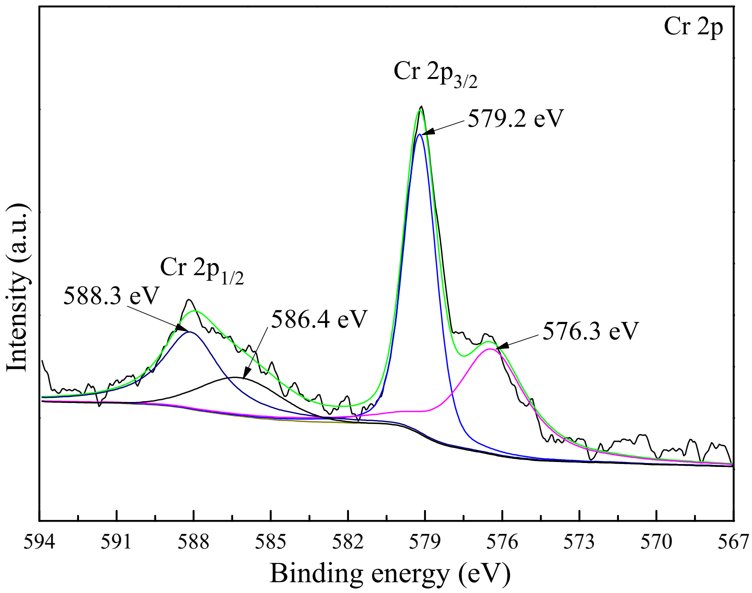

Figure S14. Cr 2p XPS spectrum of used Ag/CeO_2@C-2 after five cycle times.

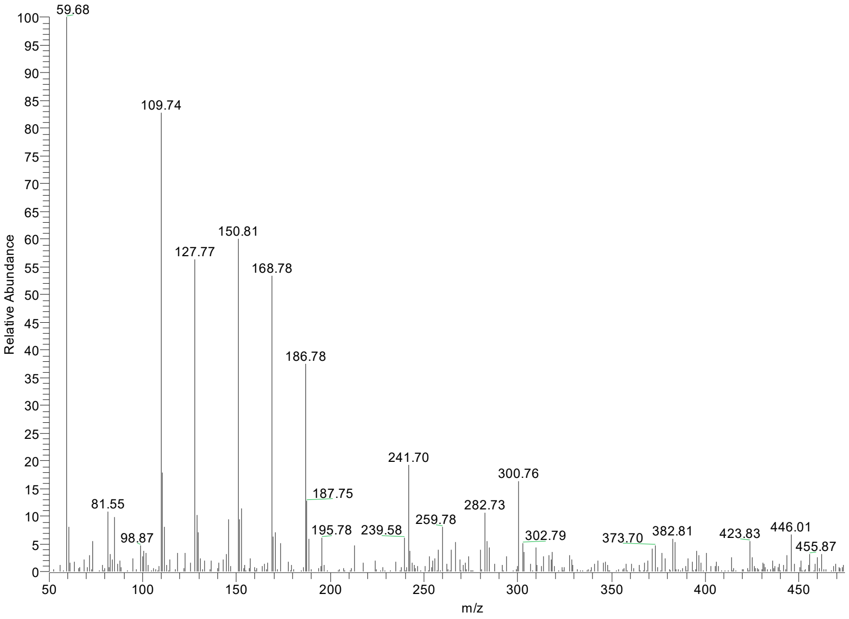

Figure S15. HPLC/MS spectrum of TCH over Ag/CeO_2@C-2 in visible light region.
